# Exploring the magnitude and drivers of the double burden of malnutrition at maternal and dyad levels in peri‐urban Peru: A cross‐sectional study of low‐income mothers, infants and young children

**DOI:** 10.1111/mcn.13549

**Published:** 2023-07-24

**Authors:** Rebecca Pradeilles, Edwige Landais, Rossina Pareja, Sabrina Eymard‐Duvernay, Oonagh Markey, Michelle Holdsworth, Emily K. Rousham, Hilary M. Creed‐Kanashiro

**Affiliations:** ^1^ Centre for Global Health and Human Development School of Sport, Exercise and Health Sciences Loughborough University Loughborough UK; ^2^ UMR MoISA (Montpellier Interdisciplinary Centre on Sustainable Agri‐Food Systems) Univ Montpellier, CIRAD, CIHEAM‐IAMM, INRAE, Institut Agro, IRD Montpellier France; ^3^ Instituto de Investigación Nutricional Lima Peru

**Keywords:** anaemia, double burden of malnutrition, infant and young children, overweight/obesity, Peru, urban, women of reproductive age

## Abstract

Multiple forms of malnutrition coexist in Peru, especially in peri‐urban areas and poor households. We investigated the magnitude of, and the contribution of, dietary and socio‐demographic factors to the double burden of malnutrition (DBM) at maternal (i.e., maternal overweight/obesity with anaemia) and dyad (i.e., maternal overweight/obesity with child anaemia) levels. A cross‐sectional survey was conducted among low‐income mother–child (6–23 months) dyads (*n* = 244) from peri‐urban communities in Peru. Dietary clusters and the minimum dietary diversity score (MDD) were generated for mothers and infants, respectively. A composite indicator using the maternal dietary clusters and the MDD was created to relate to dyad level DBM. Two dietary clusters were found: (i) the ‘high variety (i.e., animal‐source foods, fruit and vegetables), high sugary foods/beverages’ (*cluster 1*) and (ii) the ‘high potato, low fruit and vegetables, low red meat’ (*cluster 2*). DBM prevalence among mothers and dyads was 19.9% and 36.3%, respectively. Logistic regression analyses revealed that the only socio‐demographic factor positively associated with maternal DBM was maternal age (aOR/5 years: 1.35 [1.07, 1.71]). Mothers belonging to diet *cluster 1* were less likely to experience the DBM (aOR = 0.52 [0.26, 1.03]), although CIs straddled the null. Socio‐demographic factors positively associated with dyad level DBM included maternal age (aOR/5 years: 1.41 [1.15, 1.73]), and having ≥ two children under 5 years (aOR = 2.44 [1.23, 4.84]). Diet was not associated with dyad‐level DBM. Double‐duty actions that tackle the DBM are needed given that one‐third of dyads and a fifth of mothers had concurrent overweight/obesity and anaemia.

## Key messages

- Using anthropometric data and a biomarker of anaemia (haemoglobin), we found a relatively high prevalence of the double burden of malnutrition (DBM) at maternal and dyad levels in low‐income peri‐urban communities of Peru.
- There was no socioeconomic patterning in the DBM at maternal or dyad level. This indicates that a whole‐population approach should be adopted.
- Two maternal dietary clusters were identified; one cluster was considered *more* nutritious despite the presence of high sugary items in the diet (i.e., the ‘high variety [including animal‐source food, fruit and vegetables], high sugary foods and beverages’) and one *less* nutritious (i.e., the ‘high potato, low fruit and vegetables, low red meat’). The DBM was less likely in mothers belonging to the *more* nutritious cluster.
- Double‐duty actions that prioritise optimum diet quality for all should be implemented to tackle the DBM in this context.

## Graphical abstract

We investigated the magnitude of, and the contribution of, dietary and socio‐demographic factors to the double burden of malnutrition (DBM) at maternal (i.e., maternal overweight/obesity with anaemia) and dyad (i.e., maternal overweight/obesity with child anaemia) levels among low‐income mother–child dyads from peri‐urban communities in Peru. We found a relatively high prevalence of the DBM at maternal and dyad levels (19.9% and 36.3%, respectively). There was no socioeconomic patterning in the DBM. Despite weak evidence linking diet and DBM, our results suggest that the concurrence of overweight/obesity and anaemia was less likely in those who belonged to the ‘high variety, high sugary foods and beverages’ cluster compared with the ‘high potato, low fruit and vegetables, low red meat’ cluster. Given the relatively high prevalence of concurrent overweight/obesity and micronutrient malnutrition in our sample, double‐duty actions should prioritise optimum diet quality for all.

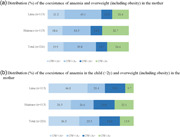

## 1 INTRODUCTION

According to the World Health Organization (WHO), malnutrition is defined as ‘deficiencies or excesses in nutrient intake or imbalance of essential nutrients’ (World Health Organization, 2023). The double burden of malnutrition (DBM) refers to both undernutrition (wasting, stunting, underweight and micronutrient deficiencies), overweight/obesity and diet‐related noncommunicable diseases (World Health Organization, 2023). Undernutrition and overweight/obesity are not only an epidemiologic phenomenon but are also biologically interlinked (Wells et al., 2020). Furthermore, both are driven by common underlying factors (i.e., shared drivers), such as early life nutrition, diet quality, food environments and socioeconomic factors that represent common opportunities for double impact (Hawkes et al., 2020; Pradeilles et al., 2019). The need to reshape interventions to simultaneously address multiple forms of malnutrition via double‐duty actions has been recently highlighted (Hawkes et al., 2020).

Over the last 30 years, Peru has made significant progress in reducing undernutrition, with the prevalence of stunting among children under 5 years decreasing from 36.6% in 1992 (Instituto Nacional de Estadistica e Informatica (INEI), 1992) to 11.5% in 2020 (Instituto Nacional de Estadistica e Informatica [INEI], 2020). However, this decrease is not equally distributed and varies according to urbanicity. Indeed, urban stunting decreased at a faster pace compared to rural stunting (Santos et al., 2021). Despite marked improvements in stunting reduction, the prevalence of iron‐deficiency anaemia in women of reproductive age (WRA) and infants and young children (IYC) remains high. Indeed, recent estimates indicate that the prevalence of iron‐deficiency anaemia among children aged 6–36 months is 38.8%, while in WRA it is estimated at 18.8% (Instituto Nacional de Estadistica e Informatica [INEI], 2021). Additionally, with increasing economic development and urbanisation, there has been a rapid increase in overweight/obesity prevalence in adults. Between 2008 and 2019, overweight (BMI 25.0–29.9 kg/m^2^) in WRA increased from 34.0% to 39.5% and obesity increased from 14.1% to 23.4% (Instituto Nacional de Estadistica e Informatica [INEI], 2021; Poterico et al., 2012). Obesity among women in the relatively poorest group and in those living in rural areas increased at a faster rate compared to their counterparts (Santos et al., 2021). Contrary to what is reported for WRA, overweight/obesity in children under 5 years has slightly decreased over time, from 11.8% in 2000 to 9.6% in 2021 (Instituto Nacional de Estadistica e Informatica [INEI], 2021). However, overweight/obesity in children 5–9 years is considerably higher (37.4% in 2027–2018) (Ministerio de Salud, 2021) indicating that the increase in prevalence occurs from early childhood. Increases in overweight/obesity prevalence may partly be due to changes in food consumption patterns. According to a recent household food expenditure survey conducted in Peru between 2001 and 2017, the energy from unhealthy foods that are usually energy‐dense and nutrient‐poor (e.g., sugar‐sweetened beverages, chocolate or cakes) increased at the expense of energy from healthy nutrient‐dense foods, such as fruits or vegetables (Santos et al., 2021).

With a high prevalence of anaemia in both children and WRA, a high prevalence of overweight/obesity in WRA and a significant prevalence of stunting among children under 5 years, Peru is experiencing the coexistence of multiple forms of malnutrition at population, household and individual levels (Curi‐Quinto et al., 2019; Mendoza‐Quispe et al., 2021; Pomati et al., 2021; Santos et al., 2021). This burden of malnutrition may potentially cause both economic losses and increasing health care costs, the latter resulting from higher risks of developing co‐morbidities such as type 2 diabetes (Nugent et al., 2020). This calls for action addressing multiple forms of malnutrition in this context.

Most studies conducted in Peru have used nationally‐representative surveys to describe trends in the prevalence of coexisting forms of malnutrition at either population (Curi‐Quinto et al., 2019; Santos et al., 2021), household (Curi‐Quinto et al., 2019; Mendoza‐Quispe et al., 2021; Pomati et al., 2021; Santos et al., 2021) or individual level (Irache et al., 2022a). However, most of the studies investigating factors associated with the DBM in Peru have only focused on anthropometric status of individuals and not on biomarkers of micronutrient status such as anaemia, and none of them have investigated diet as a potential shared driver (Curi‐Quinto et al., 2019; Mendoza‐Quispe et al., 2021; Santos et al., 2021). A recent study in Peru on social patterning of malnutrition reported that household level DBM was more likely in poorer households, in those living in less urbanised areas and in those where the mother was less educated (Mendoza‐Quispe et al., 2021). The aims of the present study were to investigate, in low‐income peri‐urban communities of Peru, the magnitude of, and the contribution of dietary and socio‐demographic factors to the DBM at maternal (i.e., maternal overweight/obesity and anaemia) and dyad (i.e., maternal overweight/obesity and child anaemia) levels.

## 2 METHODS

### 2.1 Study design and sampling

The PERUSANO cross‐sectional survey was conducted among low‐income mother–child dyads in Peru, from December 2019 to March 2020. It took place in two peri‐urban communities: one in Lima (Manchay) and one in Huánuco district (Huánuco; Andean highlands ~1900 m above sea level). This survey is part of a wider mixed‐method interdisciplinary project that aims to address the DBM in Peru. The first phase (formative research) aimed to characterise the burden of malnutrition and associated factors as well as the challenges and facilitators of healthy infant and young child feeding practices to inform the second phase of the project that incorporates a codesign approach with community stakeholders (health care professionals, mothers and family caregivers, and other community actors) to scope opportunities to address the identified challenges.

The target sample size was 360 mother–child dyads. A purposive quota sampling was employed to recruit mothers with children aged 6–23 months, with equal numbers across age groups (6–11, 12–17 and 18–23 months) and study site (Lima/Huánuco). In each study area, we purposively selected the principal health centre and one subsidiary health centre. Peri‐urban communities under the jurisdiction of these health centres were then selected to participate. Enumerators mapped each block of houses within each ‘sector’ (local planning administrative unit for urban areas) of the community within the health centre catchment, and the block was used as the sampling unit. We chose a block at random as the starting point from the mapped sector, then proceeded to knock on the first house, and every third house thereafter until completing the sector. A random starting point was chosen for the next sector, and recruitment continued. Recruitment stopped in March 2020 due to the COVID‐19 pandemic, at which point 244 mother–child dyads had been recruited and no further recruitment took place.

A screening questionnaire was used to check eligibility of mothers and IYC with the following inclusion criteria: (i) singleton infants aged ≥6 months and <24 months on the day of interview; (ii) no congenital malformations affecting nutrition or growth; and (iii) primary residence of mother/primary caregiver at the study site for the 6 months preceding the interview.

### 2.2 Data collection

Data were collected using structured questionnaires with pre‐coded responses, administered by trained enumerators. Interviews were conducted face‐to‐face at participants’ households. The questionnaire was produced in English and translated to Spanish and checked for accuracy by a team member fluent in both languages. Questionnaires (*n* = 20) were piloted in Lima with mothers of IYC living in Canto Grande, a similar community to the target communities. Data were collected on paper and then entered in Microsoft Access. Automated consistency checks were run and double data entry for a random sample of 10% of questionnaires was used to calculate the error rate. A pre‐defined threshold was set to determine an acceptable level of variation in responses, with the error rate set at 1%.

Data collected included household socio‐demographic characteristics, maternal qualitative food frequency questionnaire (FFQ) over the last 7 days (*n* = 26 food groups) adapted from a previous study conducted in Peru (Alae‐Carew et al., 2020), child quantitative 24‐h dietary recall, capillary blood sample, weight and height/length (see 10.17028/rd.lboro.18750458 for full version of the questionnaire). Haemoglobin (Hb) concentration in capillary blood was measured using Hemocue HB201 in both mothers and IYC. For mothers, weight was measured using digital portable scales (Seca 803) accurate to 100 g, and height was measured to the nearest millimetre using a portable stadiometer (Seca 213). For IYC, weight was measured with paediatric digital scales (Seca 354) accurate to 10 g and length was measured using a lightweight infantometer to the nearest millimetre (Perspective Enterprises).

### 2.3 Data management and analyses

#### 2.3.1 Anthropometric and haemoglobin outcomes

The two outcomes of interest were maternal level DBM (defined as mothers who had both overweight/obesity and anaemia) and dyad level DBM (defined as a mother with overweight/obesity whose child aged 6–23 months had anaemia).

Maternal body mass index (BMI) was assessed from measured weight and height, and classified into four groups based on the WHO cut‐offs: <18.5 kg/m^2^ underweight, 18.5–24.9 kg/m^2^ healthy weight, 25.0–29.9 kg/m^2^ overweight, and ≥30 kg/m^2^ obese (World Health Organization, 1995). IYC length‐for‐age (LAZ) and weight‐for‐length *z*‐scores (WLZ) were calculated according to the World Health Organization, 2006 Child Growth Standards (World Health Organization, 2006). Stunting was defined by LAZ < −2 *z*‐scores, wasting was defined by WLZ < −2 *z*‐scores, pre‐overweight was defined by WLZ ≥ 1 *z*‐score and overweight/obesity was defined by WLZ ≥ 2 *z*‐scores (World Health Organization, 2006).

To define anaemia in mothers and IYC, the WHO Hb cut‐offs for nonpregnant women and children aged 6–59 months were used, respectively (World Health Organization, 2011). Thus, mothers with Hb <120 g/L and IYC with Hb <110 g/L were considered as anaemic. Data were adjusted by −7 g/L for altitude in Huánuco (World Health Organization, 2011).

#### 2.3.2 Dietary factors

For the children, the dietary diversity score (DDS) (World Health Organization and the United Nations Children's Fund (UNICEF), 2021) was generated using mother/caregiver reported intakes of foods and beverages during the past 24 h. IYC who consumed at least five out of the eight pre‐defined food groups were classified as meeting the minimum dietary diversity (MDD) (World Health Organization and the United Nations Children's Fund (UNICEF), 2021). The MDD is a validated population‐level indicator of micronutrient adequacy of the diet in IYC (World Health Organization, 2008).

For the mother, 7‐day FFQ was used to generate dietary clusters. Frequencies of consumption were first converted from weekly to daily frequencies. Given the relatively small number of participants in the study and the low frequencies of consumption on some particular food groups, the 26 food groups were re‐categorised into 10 food groups. These included (i) grains; (ii) dairy products; (iii) red meat and offal; (iv) processed meat, pizza, deep fried foods and burgers; (v) poultry, fish, seafood, eggs; (vi) vegetables; (vii) legumes; (viii) potatoes and other tubers; (ix) fruits; and (x) sweet foods and beverages. The grouping was primarily informed by the nutrition composition of items (nutrient content and usage) but also by their relevance to the DBM (overweight/obesity and anaemia) and the frequencies of consumption. To account for the skewness in the distribution of the frequency of consumption and the fact that more than 50% of individuals were non‐consumers on some food groups, a binary variable was created to categorise individuals consuming below or above the median frequency of consumption for each food group. The latter was used as input variable to run the multiple correspondence analysis (MCA). MCA and cluster analysis, known as data‐driven approaches, were used in combination to obtain dietary patterns and groups of individuals (i.e., clusters) based on these patterns (Greenacre, 1992; Greenacre & Blasius, 2006). MCA was first used as a dimensionality reduction technique to obtain a smaller number of dimensions (i.e., patterns) while retaining as much of the variability in the original data as possible. The number of dimensions to retain (*n* = 3) was based on breaks in the scree plot and cumulative percent of inertia (58.9%). The Ward's hierarchical clustering analysis was then conducted on the three dimensions retained from the MCA to organise participants into groups of similar individuals with respect to dietary patterns. A two‐cluster‐based solution was selected, established on statistical criteria and interpretability (Supporting Information: Figure 1).

The maternal dietary clusters were used as the main exposure when investigating the relationship between diet and maternal level DBM. For the models using dyad level DBM as a main outcome, we created a composite dietary indicator using the maternal dietary clusters and the MDD indicator. Mother–child pairs with a relatively *healthier* diet (i.e., healthier maternal dietary cluster and child MDD met) were compared to those with a *less healthy* diet (i.e., any other combinations, e.g. healthier maternal cluster but child MDD not met).

#### 2.3.3 Socio‐demographic factors

A common household wealth index was created using factor analysis (i.e., MCA) applied to proxy indicators of the household environment (ownership of consumer durables; source of drinking water and type of toilet facilities; number of household members per room used for sleeping; type of materials used for the floors, roof and walls; and livestock ownership). The household wealth index as a continuous score was split into tertiles of socioeconomic status (SES), with the first tertile representing the relatively poorest households. The computation of the scores has been described in more detail elsewhere (Pradeilles et al., 2022). Mothers’ self‐report of completed educational level was categorised into three groups: less than secondary, secondary/technical and university level. Other socio‐demographic characteristics included maternal working status (yes/no), marital status (married/living together vs. not), child's age and sex, maternal age and place of residence (Lima vs. Huánuco).

### 2.4 Statistical analyses

Descriptive statistics (mean, SD or number (*n*), percent) were generated for socio‐demographic characteristics, anthropometric and biological status of mothers and infants. Chi‐square tests and *t*‐tests were used for area comparisons and cluster comparisons for categorical and continuous variables, respectively. Univariable and multivariable logistic regression models were used to investigate potential socio‐demographic and dietary factors associated with maternal and dyad level DBM. Directed acyclic graphs (DAGs) (i.e., hypothesised models of pathways related to the outcome) informed the model‐building process (see Supporting Information: Figures 2 and 3 for DAGs for maternal and dyad level DBM). DAGs are causal diagrams that provide a method for visualising relationships between variables to build unbiased (or less biased) causal models (Greenland et al., 1999; Moodie & Stephens, 2010). Statistical analyses were undertaken using Stata SE version 17.

### 2.5 Ethical statement

Ethical approval for this study was obtained from the Ethical Review Committee of the Instituto de Investigación Nutricional (IIN) Peru (reference 388‐2019/CIEI‐IIN) and Loughborough University (C19‐87). Written informed consent was provided by all participants after receiving written and verbal information about the study. Participants were informed of the right to withdraw from participation at any stage.

## 3 RESULTS

### 3.1 Sample characteristics

The mean age of mothers and IYC was 29.5 (7.0) years and 15.0 (5.3) months, respectively, with no differences between Lima and Huánuco (Table 1). More than half of the mothers (56.6%) had a secondary/technical education and a significantly higher proportion of mothers from Huánuco than in Lima attended university (16.8% vs. 4.8%; *p* = 0.002). One‐third of the mothers worked (32.4%), the large majority were married or lived with their partners (86.5%). Mothers had, on average, 2.3 (1.5) children. A significantly greater proportion of mothers from Huánuco belonged to the low socioeconomic group (43.7% vs. 23.2%; *p* < 0.001). Proxy indicators of the household environment stratified by area of residence are presented in Supporting Information: Table 1.

**Table 1:** Characteristics of mother/infant dyads, by area of residence.

	Total (*n* = 244)	Lima (*n* = 125)	Huánuco (*n* = 119)	
	Mean (SD)/*n* (%)	Mean (SD)/*n* (%)	Mean (SD)/*n* (%)	*p* Value
*Sociodemographic characteristics*				
Mother's age at time of survey completion (years)	29.5 (7.0)	30.2 (6.7)	28.8 (7.2)	0.107
Maternal education				
<secondary	80 (32.8)	40 (32.0)	40 (33.6)	0.006
Secondary/technical	138 (56.6)	79 (63.2)	59 (49.6)	
University	26 (10.7)	6 (4.8)	20 (16.8)	
Maternal occupation (working)	79 (32.4)	40 (32.0)	39 (32.8)	0.897
Maternal marital status (married/living together)	211 (86.5)	111 (88.8)	100 (84.0)	0.277
Socioeconomic status (tertiles)				
Low	81 (33.2)	29 (23.2)	52 (43.7)	0.003
Middle	81 (33.2)	48 (38.4)	33 (27.7)	
High	82 (33.6)	48 (38.4)	34 (28.6)	
Number of children under 5 years	1.2 (0.5)	1.3 (0.6)	1.2 (0.4)	0.065
Parity	2.3 (1.5)	2.4 (1.5)	2.1 (1.5)	0.093
Child's age (months)	15.0 (5.3)	15.0 (5.2)	15.0 (5.4)	0.988
Child's sex—males (%)	129 (52.9)	63 (50.4)	66 (55.5)	0.428
*Anthropometric* a *and biological characteristics (mother)*				
Weight (kg)	60.4 (11.0)	61.2 (11.2)	59.5 (10.9)	0.224
Height (cm)	150.7 (5.2)	150.2 (5.2)	151.2 (5.2)	0.173
Body mass index (BMI) (kg/m^2^)	26.6 (4.7)	27.2 (4.8)	26.0 (4.5)	0.062
BMI categories				
Underweight (<18.5 kg/m^2^)	2 (0.9)	0 (0.0)	2 (1.8)	0.068
Normal weight (18.5–24.9 kg/m^2^)	89 (39.4)	38 (33.6)	51 (45.1)	
Overweight (25.0–29.9 kg/m^2^)	89 (39.4)	46 (40.7)	43 (38.1)	
Obese (≥30.0 kg/m^2^)	46 (20.4)	29 (25.7)	17 (15.0)	
Haemoglobin (g/L)^b^	124.3 (11.8)	122.7 (11.3)	125.9 (12.1)	0.034
Anaemia status (yes)	83 (34.0)	45 (36.0)	38 (31.9)	0.503
*Anthropometric* c *and biological characteristics (child)*				
Length‐for‐age *z*‐score < −2	21 (9.3)	12 (10.7)	9 (8.0)	0.478
Weight‐for‐length *z*‐score < −2	3 (1.3)	0 (0.0)	3 (2.7)	0.083
Weight‐for‐length *z*‐score > 1	88 (39.1)	52 (46.4)	36 (31.9)	0.025
Weight‐for‐length z‐score > 2	19 (8.4)	11 (9.8)	8 (7.1)	0.460
Haemoglobin (g/L)^b^	105.6 (10.6)	104.1 (10.3)	107.2 (10.7)	0.021
Anaemia status (yes)	150 (61.5)	89 (71.2)	61 (51.3)	0.001
*Dietary characteristics* d *(child)*				
Mean dietary diversity score (DDS)	5.7 (1.2)	5.8 (1.2)	5.7 (1.3)	0.795
Number (%) of children who met the minimum DDS (MDD)	208 (85.9)	107 (85.6)	101 (86.3)	0.871

*Note*: *T*‐test was performed for continuous variables and chi‐square for categorical/binary variables.

Data are presented as mean (SD) for continuous measures, and *n* (%) for categorical measures.

^a^ Missing data for 18 mothers.

^b^ Hb data were adjusted for altitude in Huánuco (World Health Organization, 2011).

^c^ Missing data for 19 children.

^d^ Missing data for two children.

Regarding the nutritional status of mothers, overall, almost 4 out of 10 mothers were overweight (39.4%), and one‐fifth had obesity (20.4%), with no differences according to the area of residence (Table 1). Altogether, about one‐third of mothers were anaemic (34.0%).

Regarding the nutritional status of IYC, about 10% were stunted with no differences according to the area of residence (Table 1), 39.1% were pre‐overweight, with a significantly higher proportion in Lima compared to Huánuco (46.4% vs. 31.9%, respectively; *p* = 0.025), and overall, 8.4% were overweight. Almost two‐thirds of IYC were anaemic with a large difference according to the area of residence (71.2% in Lima vs. 51.3% in Huánuco; *p* = 0.001).

### 3.2 The double burden of malnutrition among mothers and mother–child dyads

**Figure 1:**
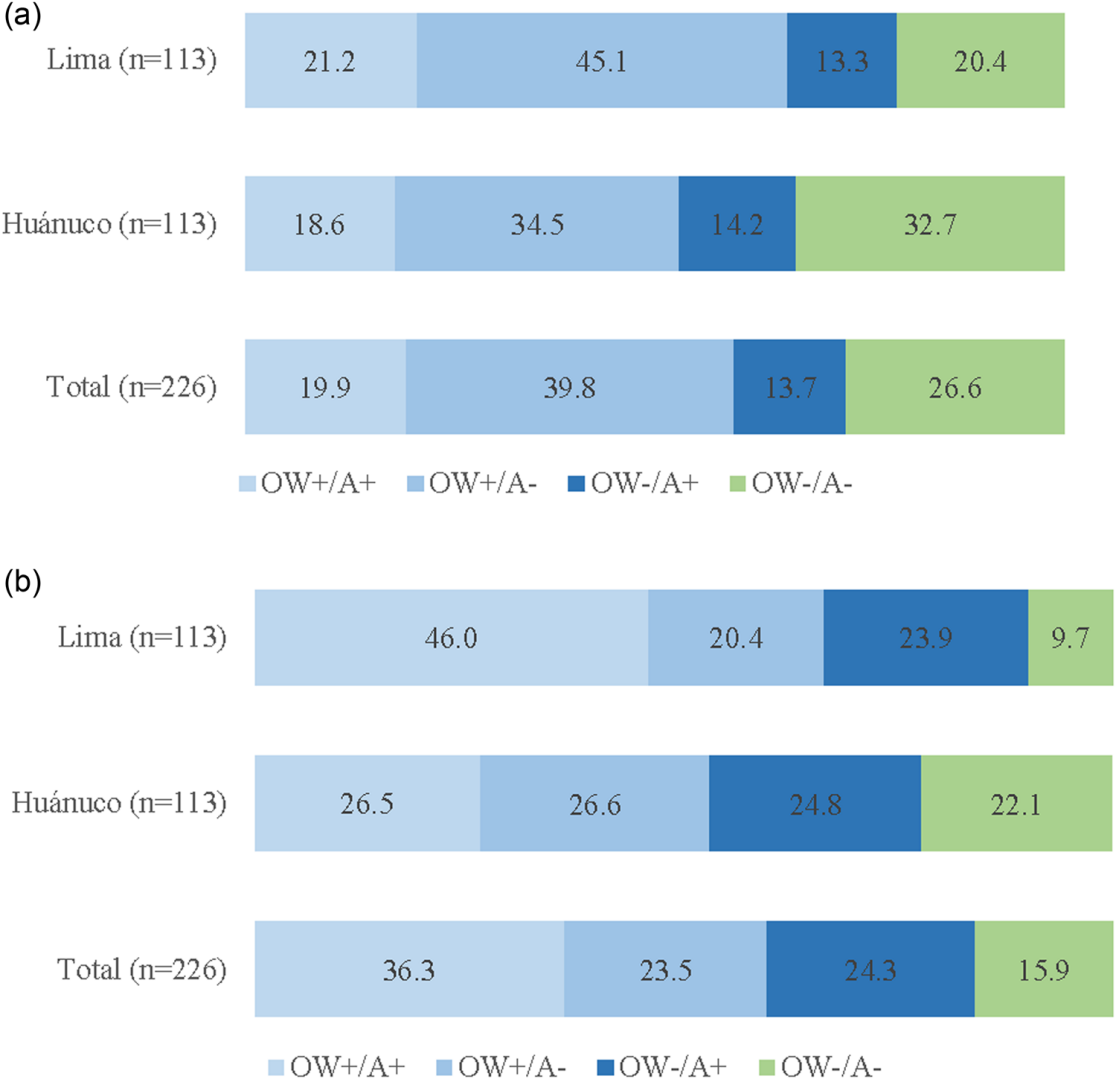
Distribution of the double burden of malnutrition at maternal (a) and dyad (b) levels. (a) Distribution (%) of the coexistence of anaemia and overweight (including obesity) in the mother. (b) Distribution (%) of the coexistence of anaemia in the child (<2 years) and overweight (including obesity) in the mother. A, anaemia; OW, overweight (including obesity).

At the maternal level, coexisting overweight and anaemia in mothers represented about one‐fifth of the sample (19.9%) and was similar according to the area of residence (Figure 1a). Most of the mothers were overweight without anaemia (39.8%) with differences according to the area of residence (45.1% in Lima vs. 34.5% in Huánuco). Overall, one‐quarter of the mothers (26.6%) did not present any coexisting forms of malnutrition with important differences between Lima and Huánuco (20.4% and 32.7%, respectively; *p* = 0.035).

At the mother–child dyad level, coexisting maternal overweight (obesity included) and child anaemia represented 36.3% of the sample (Figure 1b); 23.5% of overweight mothers had a child without anaemia; about one‐quarter (24.3%) of mothers who were not overweight had a child with anaemia, and only 15.9% of the mother–child dyads presented no coexisting forms of malnutrition. The prevalence of DBM at the dyad level was greater in Lima compared to Huánuco (46.0% vs. 26.5%, respectively; *p* = 0.002).

### 3.3 Dietary factors

The mean DDS was 5.7 (1.2) in IYC. Overall, the proportion of IYC who met the MDD was 85.9%, with no significant differences according to the area of residence (86.3% in Huánuco vs. 85.6% in Lima, *p* = 0.31).

The median daily consumption frequency of the 10 food groups for mothers is reported in Table 2. The highest median daily consumption was found for grains (2.0 [1.7–2.3]) and sweet foods and beverages (1.7 [1.0–2.9]). This was followed by fruits (1.0 [0.4–1.0]), vegetables (1.0 [0.6–1.3]) and potatoes/tubers (0.9 [0.4–1.1]). The median daily consumption of dairy, red meat and offal and of processed meat, pizza, burgers and deep‐fried foods was relatively low (<0.5 times per day).

**Table 2:** Maternal food group consumption and dietary clusters.

	Total^a^ (*n* = 243)	Cluster 1^b^ (*n* = 138)	Cluster 2^b^ (*n* = 105)	*p* Value
Grains				
Median daily frequency	2.0 (1.7–2.3)			
% above median		105 (76.1%)	77 (73.3%)	0.624
Dairy				
Median daily frequency	0.4 (0.3–0.9)			
% above median		96 (69.6%)	44 (41.9%)	<0.001
Red meat, offal				
Median daily frequency	0.3 (0.1–0.6)			
% above median		123 (89.1%)	45 (42.9%)	<0.001
Processed meat, pizza, burgers, deep‐fried foods				
Median daily frequency	0.1 (0.0–0.4)			
% above median		95 (68.8%)	73 (69.5%)	0.909
Poultry, fish, seafood, eggs				
Median daily frequency	1.3 (1.0–1.7)			
% above median		79 (57.2%)	53 (50.5%)	0.294
Vegetables				
Median daily frequency	1.0 (0.6–1.3)			
% above median		99 (71.7%)	23 (21.9%)	<0.001
Legumes				
Median daily frequency	0.3 (0.1–0.4)			
% above median		117 (84.8%)	58 (55.2%)	<0.001
Potatoes/tubers (french fries excluded)				
Median daily frequency	0.9 (0.4–1.1)			
% above median		64 (46.4%)	70 (66.7%)	0.002
Fruits				
Median daily frequency	1.0 (0.4–1.0)			
% above median		103 (74.6%)	29 (27.6%)	<0.001
Sweet foods and beverages				
Median daily frequency	1.7 (1.0–2.9)			
% above median		83 (60.1%)	41 (39.0%)	0.001

^a^ One mother with no dietary data.

^b^ Data are presented as *n* (%), *cluster 1*: ‘high variety (i.e., animal‐source foods, fruit and vegetables), high sugary foods and beverages’; *cluster 2*: ‘high potato, low fruit and vegetables, low red meat’.

Two dietary clusters emerged from the dietary clusters analysis (Table 2). In *cluster 1* relative to *cluster 2*, the proportion of consumers (i.e., mothers) above the median was higher for dairy, red meat/offal, vegetables, legumes, fruits, and sweet foods/beverages. When comparing *cluster 2* relative to *cluster 1*, the proportion of consumers above the median was higher for potatoes and tubers and notably lower for fruits, vegetables, and red meat. Therefore, we labelled *cluster 1* the ‘high variety (i.e., animal‐source foods, fruit and vegetables), high sugary foods and beverages’ cluster and *cluster 2* the ‘high potato, low fruit and vegetables, low red meat’ cluster. There were no differences between the two clusters for the consumption of ‘grains’, ‘processed meat, pizza, burgers and deep‐fried foods’ and ‘poultry, fish seafood and eggs’.

### 3.4 Factors associated with the double burden of malnutrition at maternal and dyad levels

#### 3.4.1 At maternal level

In terms of factors associated with maternal DBM, there was no evidence in support of an association between area of residence, socioeconomic status, education, parity and DBM (Table 3). The evidence in support of an association between maternal age, maternal occupation, dietary cluster and DBM was stronger. Indeed, older mothers (aOR/5 years = 1.35 [1.07, 1.71]) and working mothers (aOR = 1.86 [0.94, 3.68]) were more likely to experience maternal DBM, although the CIs straddled the null for occupation. Mothers classed in the ‘high variety, high sugary foods and beverages’ cluster had lower odds of DBM (aOR = 0.52 [0.26, 1.03]) in comparison to those in the ‘high potato, low fruit and vegetables, low red meat’ cluster, although the CIs straddled the null.

**Table 3:** Exploring factors associated with maternal‐ and dyad level double burden of malnutrition (DBM): multivariable regression models.

Factors	Maternal level DBM			Dyad level DBM		
	aOR	95% CI	*p* Value	aOR	95% CI	*p* Value
Area of residence: Huánuco (vs. Lima)	0.85	0.44, 1.63	0.617	0.42	0.24, 0.74	0.003
Household SES^a^						
Low	Ref	–	0.932	Ref	–	0.779
Middle	1.08	0.45, 2.59		1.10	0.52, 2.35	
High	0.92	0.38, 2.25		1.31	0.61, 2.82	
Maternal age (per 5 years)	1.35	1.07, 1.71	0.013	1.41	1.15, 1.73	0.001
Maternal education^b^						
<secondary	Ref	–	0.684	Ref	–	0.282
≥secondary	1.17	0.56, 2.44		0.72	0.39, 1.31	
Maternal occupation^c^ (working)	1.86	0.94, 3.68	0.073	1.73	0.96, 3.12	0.071
At least 2 children <5 years^d^	1.63	0.75, 3.54	0.217	2.44	1.23, 4.84	0.011
Parity^e^	1.20	0.91, 1.57	0.194	–	–	–
Dietary clusters^f^						
Cluster 1 ‘high variety, high sugary foods and beverages’	0.52	0.26, 1.03	0.062	–	–	–
Cluster 2 ‘high potato, low fruit and vegetables, low red meat’	Ref	_		–	–	_
Dietary indicator (proxy) for the mother/child dyad^f^						
Cluster 1 ‘high variety, high sugary foods and beverages’ + MDD child met	–	–	–	0.79	0.44, 1.42	0.426
Less optimal diet (i.e., any other combinations)	–	–	–	Ref	–	

Abbreviations: aOR, adjusted odds ratio; SES, socioeconomic status; 95% CI, confidence intervals.

^a^ Household SES adjusted for maternal age, area, maternal education, maternal occupation.

^b^ Maternal education adjusted for maternal age.

^c^ Maternal occupation adjusted for maternal age, maternal education.

^d^ At least two children <5 years adjusted for maternal age, maternal education.

^e^ Parity adjusted for maternal education and maternal age.

^f^ Dietary clusters adjusted for maternal age, area, maternal education, maternal occupation, at least 2 children <5 years, SES.

#### 3.4.2 At mother–child dyad level

With regard to factors associated with dyad level DBM, there was no evidence for an association between socioeconomic status, education and DBM (Table 3). There was stronger evidence for an association between area of residence, maternal age, occupation, number of children under 5 and DBM. Indeed, those living in Huánuco (aOR = 0.42 [0.24, 0.74]) had lower odds of experiencing dyad level DBM while older mothers (aOR/5 years: 1.41 [1.15, 1.73]), working mothers (aOR = 1.73 [0.96, 3.12]) and those with at least two children under 5 years (aOR = 2.44 [1.23, 4.84]) had higher odds of displaying dyad level DBM, although the CIs straddled the null for occupation. In addition, mother–child pairs who belonged to the *more* nutritious diet (maternal cluster 1 ‘high variety, high sugary foods and beverages’ and child MDD met) were less likely to experience DBM but the strength of the evidence for this association was weak (aOR = 0.79 [0.44, 1.42]). Univariable models for maternal and dyad level DBM are presented in Supporting Information: Table 2.

## 4 DISCUSSION

### 4.1 Summary and interpretation of findings

This study quantifies the DBM among IYC and WRA in low‐income peri‐urban communities of Peru. Furthermore, it reveals the contribution of shared dietary and socio‐demographic factors to maternal and dyad‐level DBM, and in doing so, identifies potential targets for intervention.

#### 4.1.1 Magnitude of the double burden of malnutrition among mothers and mother–child dyads

Estimates for both maternal and dyad level DBM were relatively high in the low‐income urban communities targeted. At maternal level, approximately one in five women experienced both overweight/obesity and anaemia, with no differences between Lima and Huánuco. This result indicates that the required iron intakes for some women who are overweight or obese are not met. The estimate for maternal level DBM in this sample is higher compared to the average for Peru (9.4%), for the Americas (13.5%), and compared to the pooled LMIC estimate based on 52 countries (12.4%) (Irache et al., 2022a). Evidence from LMICs suggests that the prevalence of household level DBM (defined as one or more individuals with wasting/stunting/thinness and one or more individuals with overweight/obesity within the same household) is higher in middle‐income countries like Peru, compared to low‐ or high‐income economies (Popkin et al., 2020). In our study, dyad level DBM (maternal overweight, including obesity, and child anaemia) was high (36.3%), when compared with estimates of household level DBM (defined as a mother with overweight/obesity and at least one of her children <5 years with anaemia or a mother with anaemia and at least one of her children with overweight/obesity or both combinations) for Peru (19.4%) (Irache et al., 2022b). It was also higher than estimates for the Americas region (21.6%) and for 49 LMICs combined (16.2%) (Irache et al., 2022b). The average prevalence of dyad level DBM masks important inter‐area variation in our sample (i.e., Lima [46%] and Huánuco [25%]). A recent study using data from the Peru Demographic and Health Survey (DHS) reported that between 2009 and 2016, household level DBM (defined as a child with undernutrition [stunting, underweight or wasting] and a mother with overweight/obesity) decreased from 10.7% to 7.2% (Mendoza‐Quispe et al., 2021). This decrease may partly be explained by the large reduction in absolute numbers for child undernutrition (as defined by anthropometric status) over this time period. Our estimate of dyad level DBM is five times higher than that observed in the above study—this may be due to differences in the definitions used to measure DBM (i.e., the use of biomarkers of micronutrient status over anthropometric ones for the child) and differences in sampling.

#### 4.1.2 Factors associated with the double burden of malnutrition among mothers and mother‐child dyads

##### Sociodemographic factors

A recent multi‐level analysis on global inequalities in household level DBM (i.e., maternal overweight/obesity and child stunting) conducted across 55 LMICs (1992–2018) showed that the probability of the DBM was higher among wealthier households in lower‐income countries and higher among poorer households in higher‐income countries (Seferidi et al., 2022). In our study, we did not find any socioeconomic gradient in the burden of malnutrition at maternal or dyad levels. This could be because we targeted low‐income families and hence our sample was too homogeneous to detect socioeconomic differences. Nonetheless, we did find other socio‐demographic factors associated with maternal and dyad level DBM. Indeed, older mothers were more likely to experience both maternal and dyad level DBM while mothers with at least two children under 5 were more likely to experience dyad level DBM. Those living in Huánuco versus Lima were less likely to experience dyad level DBM. This is because both maternal overweight/obesity and child anaemia were less prevalent in Huánuco.

##### Dietary factors

In both the Lima and Huánuco populations studied, more than four out of five IYC met the minimum dietary diversity. Related findings on diet quality among IYC in our sample have been published elsewhere (Pradeilles et al., 2022). We found that the proportion of children meeting the minimum meal frequency was high (~85%), as well as the proportion of IYC consuming eggs and/or flesh foods (~89%). In addition, the proportion of IYC not consuming any vegetables or fruit was very low (3.7%). These results are indicative of an overall favourable diet quality (defined according to these indicators). However, given the very high prevalence of anaemia in IYC in our survey (>60%), particularly in Lima, we can speculate that even if diets are diverse and meet the minimum number of meals to be consumed to cover energy requirements, they may not provide nutritious foods in sufficient quantities to cover key nutrient requirements. In addition, the high consumption of cereal staples in Peru may prevent absorption of key micronutrients such as iron or zinc owing to the high levels of phytates in these foods (Lönnerdal, 2000; López & Martos, 2004). As anaemia is multi‐factorial, other factors not studied here (e.g., infection, inflammation) could potentially contribute to the very high prevalence of anaemia besides micronutrient deficiencies (Chaparro & Suchdev, 2019). Indeed, we did not find any relationship between MDD and child anaemia in our survey.

We also found that the consumption of unhealthy foods and sugar‐sweetened beverages (as assessed by the 24‐h dietary recall) was high for IYC in our sample (35.5% and >78.0%, respectively) (Pradeilles et al., 2022). These foods may therefore be displacing more nutritious foods and hence contributing to micronutrient deficiencies (Lutter et al., 2021). In mothers, we identified two dietary clusters; one considered *more* nutritious despite the presence of high sugary items in the diet (i.e., the ‘high variety, high sugary foods and beverages’) and one considered *less* nutritious (i.e., the ‘high potato, low fruit and vegetables, low red meat’). While we did not find the dichotomy of clusters (healthy/prudent vs. unhealthy/western) often reported in the literature on dietary patterns in LMICs such as Brazil (Monteiro Dos Santos et al., 2021) and Thailand (Shi et al., 2018), we did find some similarities with a recent study conducted in Peru among adults aged ≥35 years (Alae‐Carew et al., 2020). Across four urban and rural study sites (sea‐level and high‐altitude), the authors found four dietary patterns classed according to stages of the nutrition transition: ‘*Stage 1*, traditional diet with high‐starch and low fat foods with low diversity; *Stage 2*, traditional diet with increasing range of high‐fibre foods as well as high‐fat foods consumed; *stage 3*, higher in processed foods and animal products, with less of the traditional high‐fibre foods; and *stage 4* (the fully transitioned diet), high diversity of food group consumption including high‐fibre, high‐fat and high‐sugar foods‘. In urban Lima, three out of five adults belonged to the *stage 4* dietary pattern while in urban Puno (highlands), 52.1% of the sample were classed in *stage 2*. In peri‐urban Tumbes (sea‐level), 81.4% of the population belonged to the *stage 3* dietary pattern. In our study, there were no socio‐demographic characteristics associated with the dietary clusters, apart from area of residence (data not shown). Indeed, the ‘high variety, high sugary foods and beverages’ diet in the present study, corresponding to the fully transitioned diet in Alae‐Carew et al. (2020), was more common in Huánuco while the ‘high potato, low fruit and vegetables, low red meat’ (closer to the traditional diet described in Alae‐Carew et al., 2020) was more frequent in Lima.

When studying the relationship between diet and different forms of malnutrition, we found that maternal anaemia (which was more common in Lima) was more likely in mothers who belonged to the ‘high potato, low fruit and vegetables, low red meat’ cluster but found no associations with maternal overweight/obesity. This aligns with findings from Alae‐Carew et al. (2020) which showed that adherence to a more traditional diet was associated with lower odds of type 2 diabetes, hypertension and high BMI. The associations between diet and either maternal or dyad level DBM were weak, although the evidence pointed towards the *more* nutritious cluster/combination being associated with lower odds of DBM.

### 4.2 Strengths and limitations

To our knowledge, the present study is one of the first studies in Peru to investigate the DBM defined using a biomarker of anaemia (Hb) and anthropometric status (overweight/obesity). Furthermore, it adds to the current body of evidence by using dietary cluster analysis to investigate diet as a potential shared driver of the DBM. Finally, it focuses on low‐income urban communities which are under‐studied. Rapid urbanisation in Peru means that peri‐urban populations like the ones in our study are expanding and individuals are living in increasingly precarious settlements. Hence it is important to understand nutritional issues and solutions in these underrepresented groups. We acknowledge several limitations that should be considered when interpreting our findings. First, our survey was cross‐sectional, which means that it cannot be used for causal inferences. Second, our sample may not be representative of all low‐income, peri‐urban populations of Peru. Selection of the study areas was done through consultation with the local and municipal authorities to target disadvantaged sectors. While there would have been some socioeconomic variation within these peri‐urban communities, the broader socioeconomic position, housing, access to health facilities and other infrastructure resources were similar. While we did not include indicators of SES in the screening questionnaire, by targeting peri‐urban areas in our study, we knew that we would target the lowest income residents. Furthermore, our sample was relatively small. Using a post hoc power calculation with *n* = 244 and a prevalence of dyad level DBM at 36%, we estimate the prevalence of dyad level DBM with a precision of 6.02%. We estimate the prevalence of maternal DBM (19.9%) with a precision of 5.01%. In addition to increasing the precision with which we could have estimated the prevalence of DBM, a larger sample size would also have resulted in more precise beta estimates for the factors associated with DBM. This may have impacted findings on the associations between shared drivers and DBM. However, our use of DAGs to identify the minimally sufficient set of covariates to adjust for, resulted in more parsimonious regression models and thus the preservation of power. Third, we used Hb measurement to define anaemia, but we acknowledge that anaemia is a multi‐factorial condition and therefore other factors we did not study could contribute to the high prevalence of anaemia. Fourth, we were unable to use the same dietary measurement in mothers and IYC. For the maternal dietary clusters, we used data from a 7‐day qualitative FFQ which was deemed more appropriate than the 24‐h dietary recall when generating dietary clusters. For IYC, we used data from a 24‐h dietary recall hence the use of MDD as a marker of diet quality. In addition, quantitative estimates of dietary intake may have aided further understanding of the high prevalence of anaemia in our sample.

## 5 CONCLUSION

The present study has highlighted a relatively high prevalence of the DBM at maternal and dyad levels in low‐income peri‐urban communities of Peru. There was no socioeconomic patterning in the DBM at both maternal and dyad levels, which indicates that a whole population approach should be adopted in the targeted urban poor communities. Despite weak evidence linking diet and DBM, our results suggest that the concurrence of overweight/obesity and anaemia was less likely in those who belonged to the ‘high variety, high sugary foods and beverages’ cluster compared with the ‘high potato, low fruit and vegetables, low red meat’ cluster. Given the relatively high prevalence of concurrent overweight/obesity and micronutrient malnutrition in our sample, double‐duty actions should prioritise optimum diet quality for all. Indeed, interventions should focus on promoting healthy diets that are nutrient‐rich and on limiting the consumption of energy‐dense, nutrient‐poor foods and beverages. Addressing food system challenges to ensure healthy diets for all is therefore of paramount importance.

## AUTHOR CONTRIBUTIONS

Emily K. Rousham, Rossina Pareja, Hilary M. Creed‐Kanashiro, Rebecca Pradeilles, Oonagh Markey and Michelle Holdsworth designed the study. Emily K. Rousham, Rossina Pareja, Hilary M. Creed‐Kanashiro, Rebecca Pradeilles, Michelle Holdsworth and Edwige Landais were involved in designing the data collection approach and tools. Rossina Pareja and Hilary M. Creed‐Kanashiro led data collection and performed data quality checks. Sabrina Eymard‐Duvernay, Rebecca Pradeilles and Edwige Landais managed and analysed the data. Rebecca Pradeilles and Edwige Landais wrote the first draft of the paper. Emily K. Rousham, Michelle Holdsworth, Hilary M. Creed‐Kanashiro, Rossina Pareja, Oonagh Markey and Sabrina Eymard‐Duvernay provided critical input to the manuscript. All authors approved the final version.

## CONFLICT OF INTEREST STATEMENT

The authors declare no conflict of interest.

## Supporting information

Supporting information.

## Data Availability

The data that support the findings will be available on the Loughborough University Research Repository at doi:10.17028/rd.lboro.c.6329105 (The PERUSANO quantitative survey) following a period of embargo to allow for completion of research and publication of findings.
