# Supplementary material for: Exploring the magnitude and drivers of the double burden of malnutrition at maternal and dyad levels in peri‐urban Peru: A cross‐sectional study of low‐income mothers, infants and young children
Source: Matern Child Nutr. 2023 Jul 24;19(4):e13549. doi: 10.1111/mcn.13549 (PMC10483951; doi:10.1111/mcn.13549)
Supplement: Supplementary file 1 — Supporting information. [file MCN-19-e13549-s001.docx]

**Exploring the magnitude and drivers of the double burden of malnutrition at maternal and dyad levels in peri-urban Peru: a cross-sectional study of low-income mothers, infants and young children**

**Pradeilles R, Landais E, et al.**

Online Supplementary Material

**Supplemental Figure 1**. Dendrogram for cluster analysis

**Supplemental Figure 2**. Directed acyclic graphs (DAG) exploring the relationship between explanatory factors and individual level double burden of malnutrition (DBM)

*Model 1: DAG exploring the relationship between area of residence and maternal DBM*


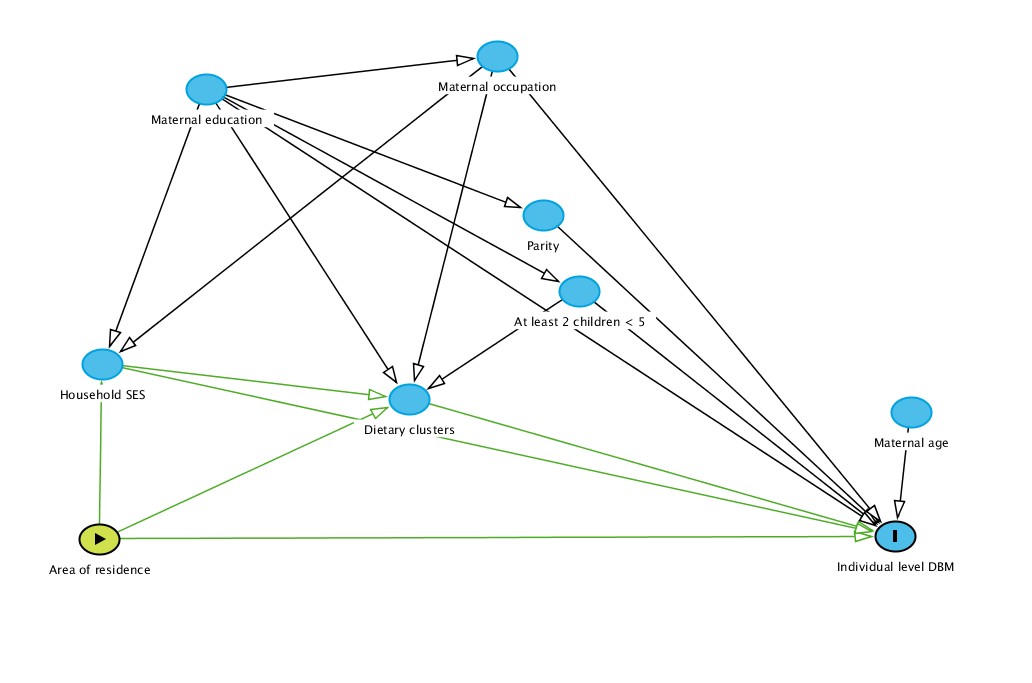


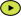
exposure
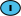
outcome
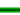
causal path
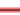
biasing path
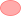
ancestor of exposure and outcome
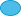
ancestor of outcome
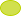
ancestor of exposure
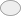
 unobserved

*Model 2: DAG exploring the relationship between household SES and maternal DBM*


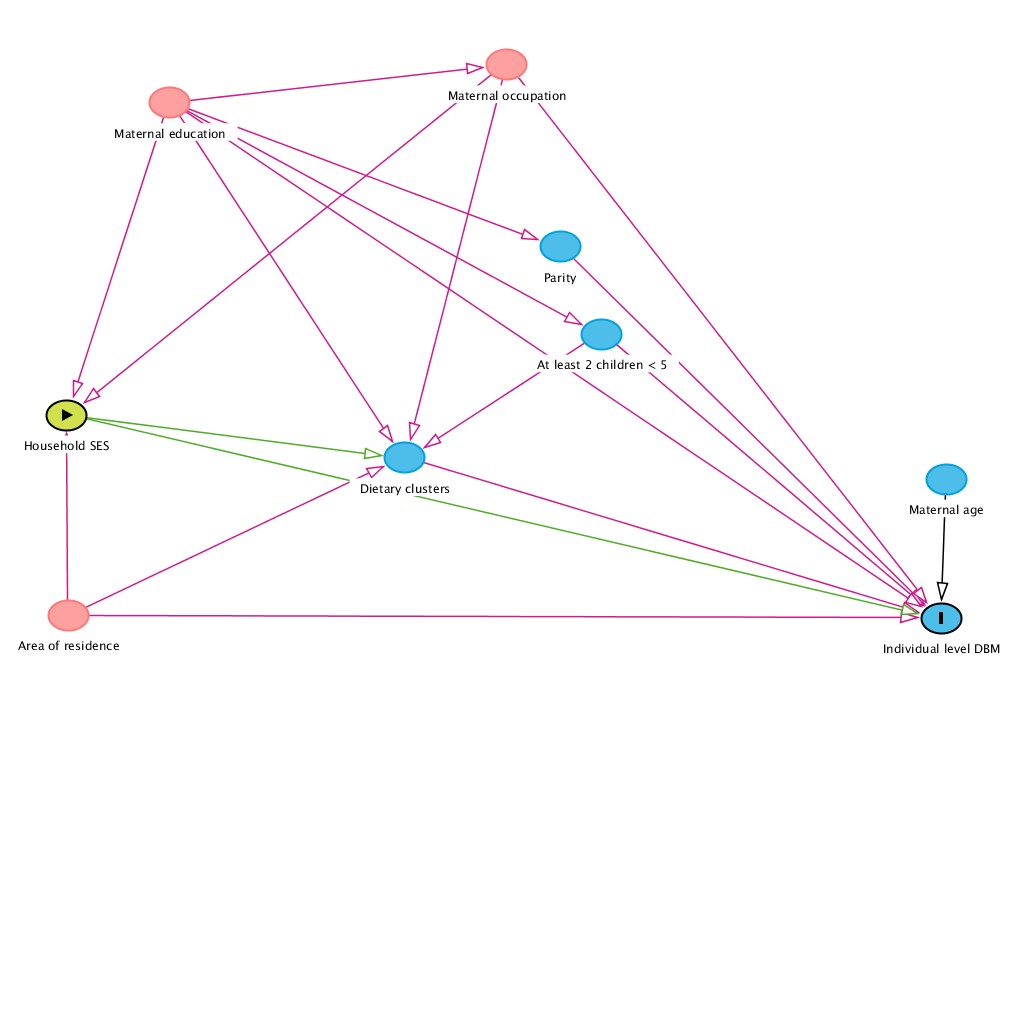


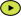
exposure
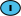
outcome
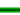
causal path
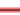
biasing path
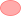
ancestor of exposure and outcome
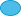
ancestor of outcome
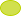
ancestor of exposure
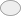
 unobserved

*Model 3: DAG exploring the relationship between maternal age and maternal DBM*


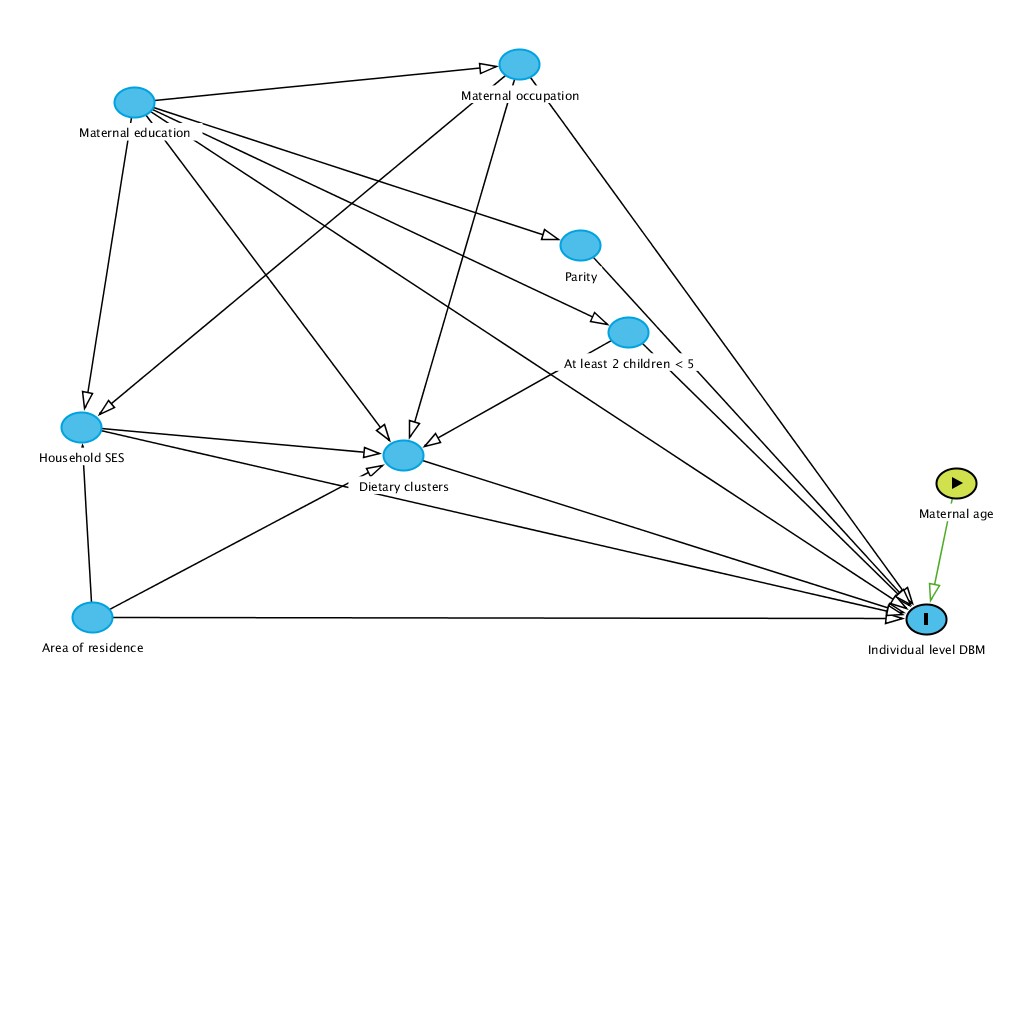


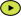
exposure
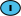
outcome
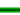
causal path
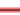
biasing path
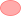
ancestor of exposure and outcome
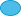
ancestor of outcome
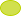
ancestor of exposure
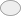
 unobserved

*Model 4: DAG exploring the relationship between maternal education and maternal DBM*


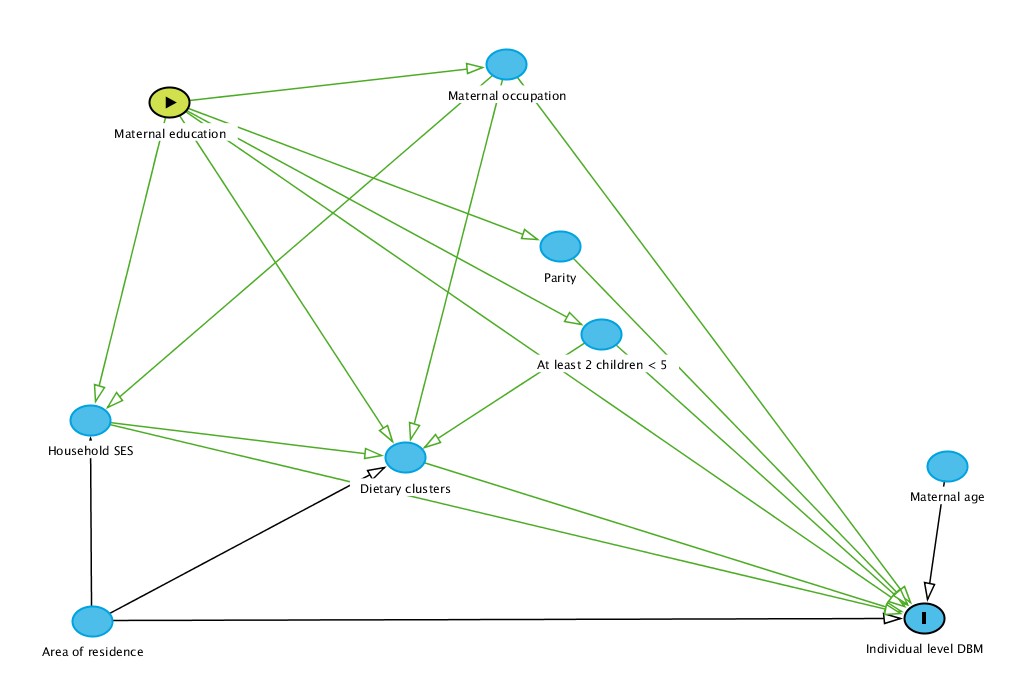


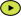
exposure
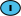
outcome
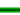
causal path
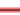
biasing path
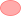
ancestor of exposure and outcome
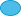
ancestor of outcome
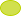
ancestor of exposure
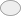
 unobserved

*Model 5: DAG exploring the relationship between maternal occupation and maternal DBM*


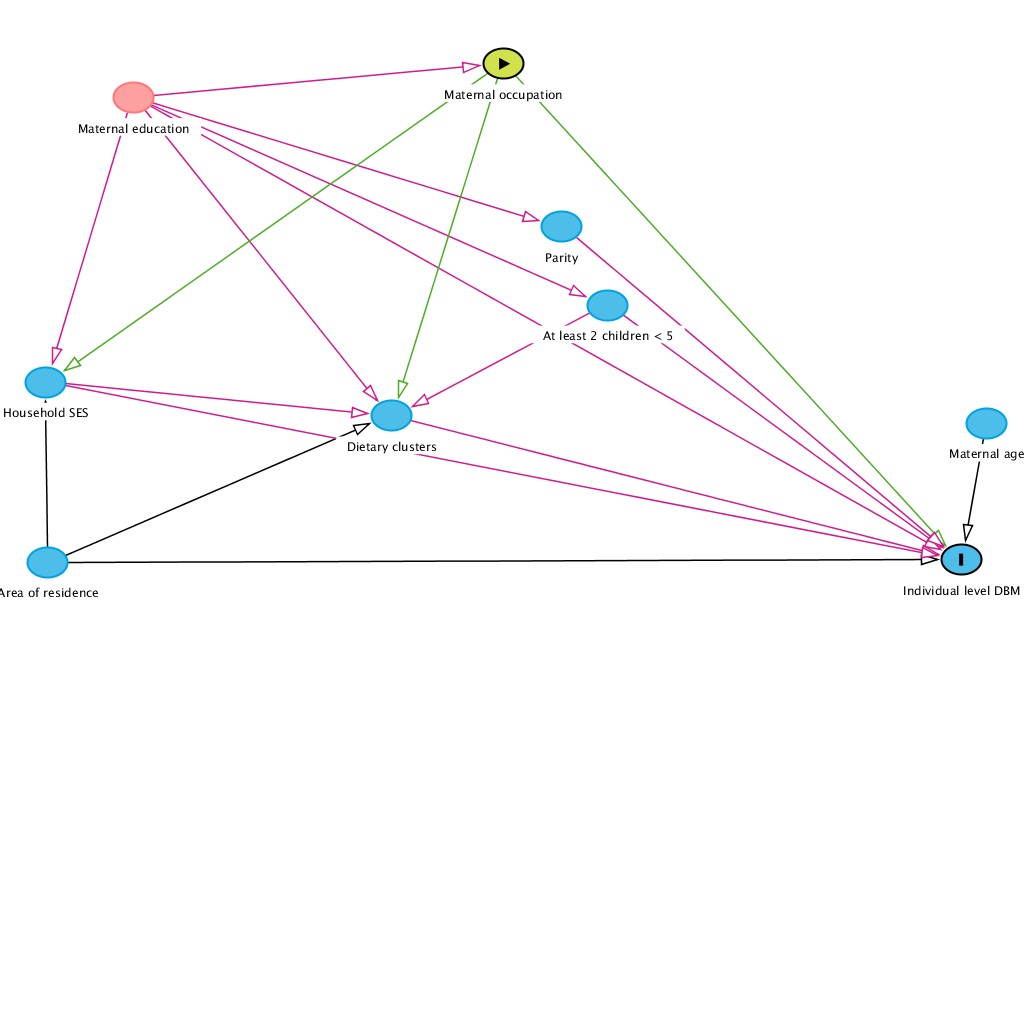


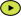
exposure
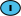
outcome
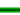
causal path
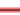
biasing path
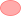
ancestor of exposure and outcome
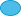
ancestor of outcome
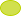
ancestor of exposure
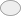
 unobserved

*Model 6: DAG exploring the relationship between the number of children under five and maternal DBM*


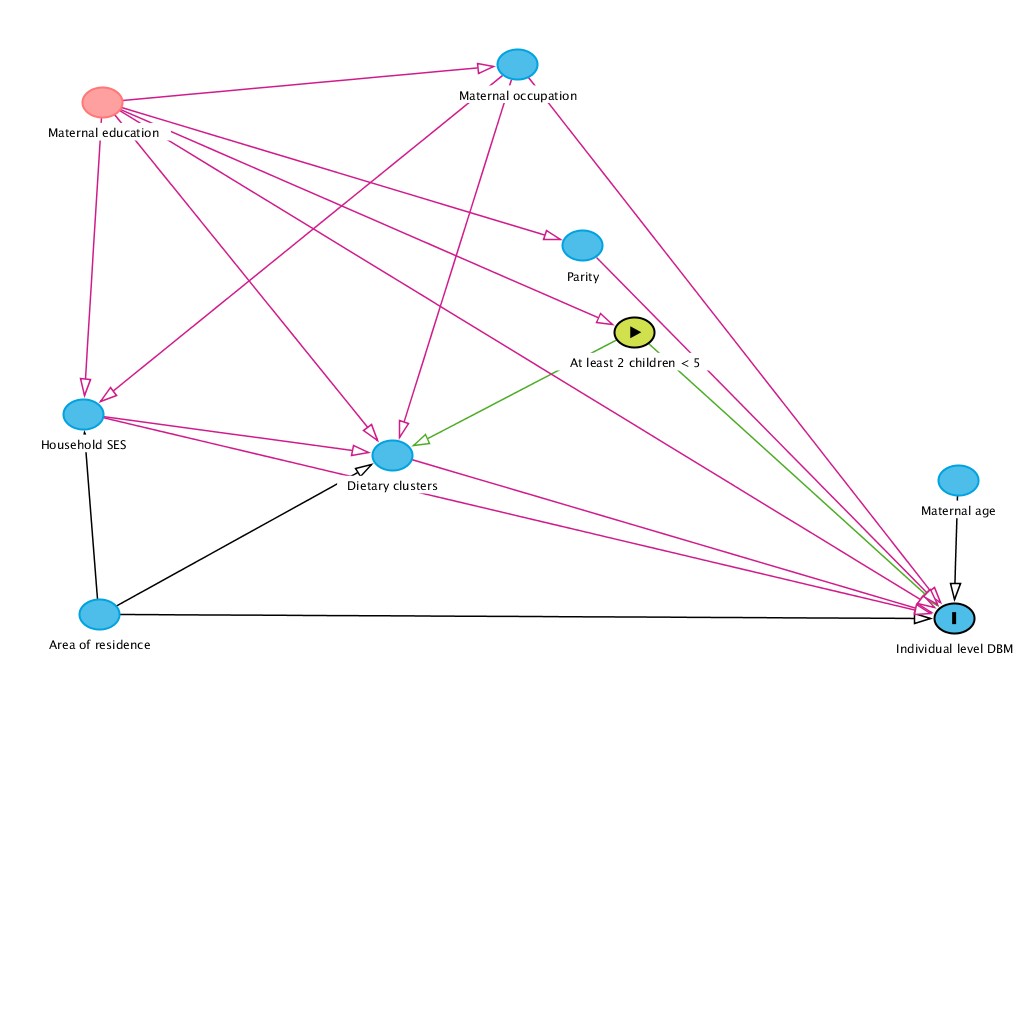


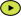
exposure
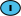
outcome
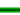
causal path
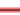
biasing path
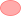
ancestor of exposure and outcome
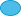
ancestor of outcome
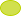
ancestor of exposure
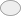
 unobserved

*Model 7: DAG exploring the relationship between parity and maternal DBM*


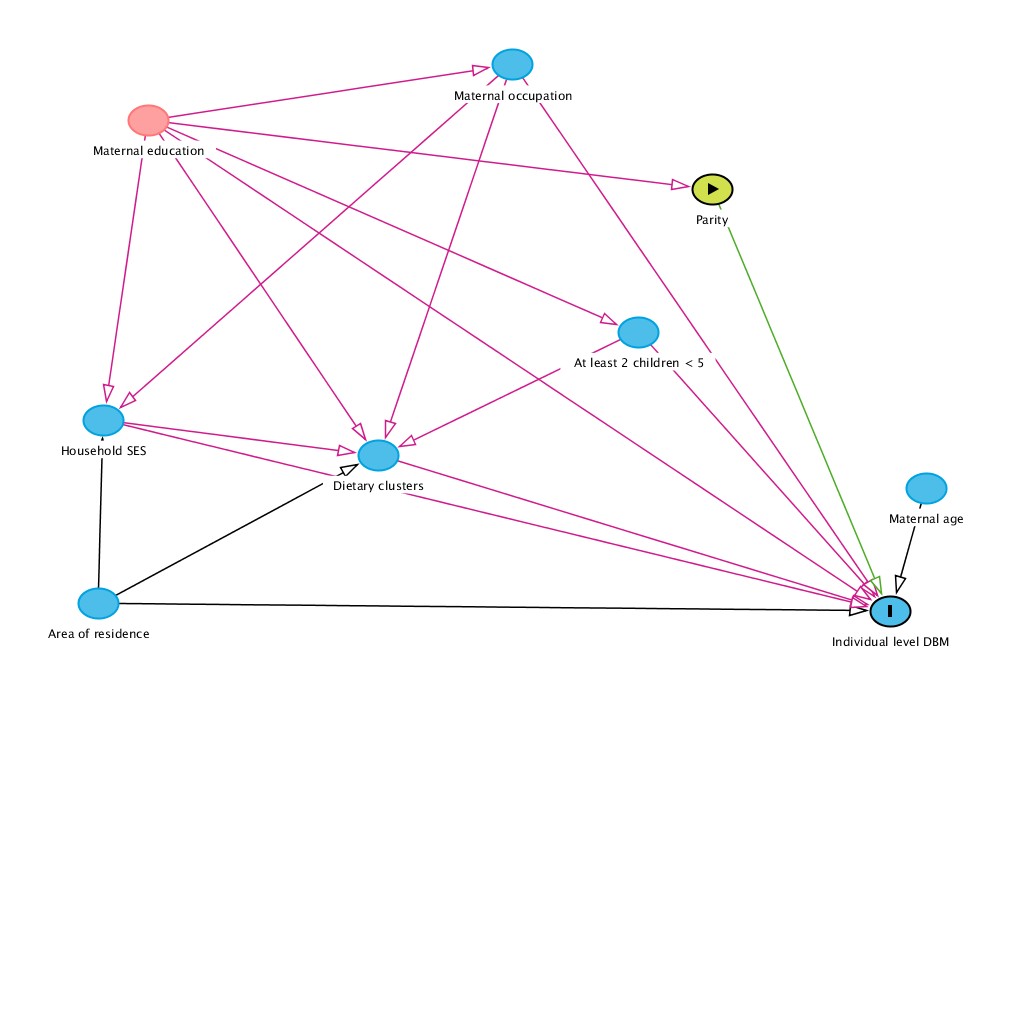


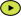
exposure
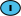
outcome
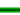
causal path
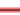
biasing path
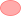
ancestor of exposure and outcome
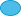
ancestor of outcome
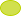
ancestor of exposure
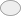
 unobserved

*Model 8: DAG exploring the relationship between dietary clusters and maternal DBM*


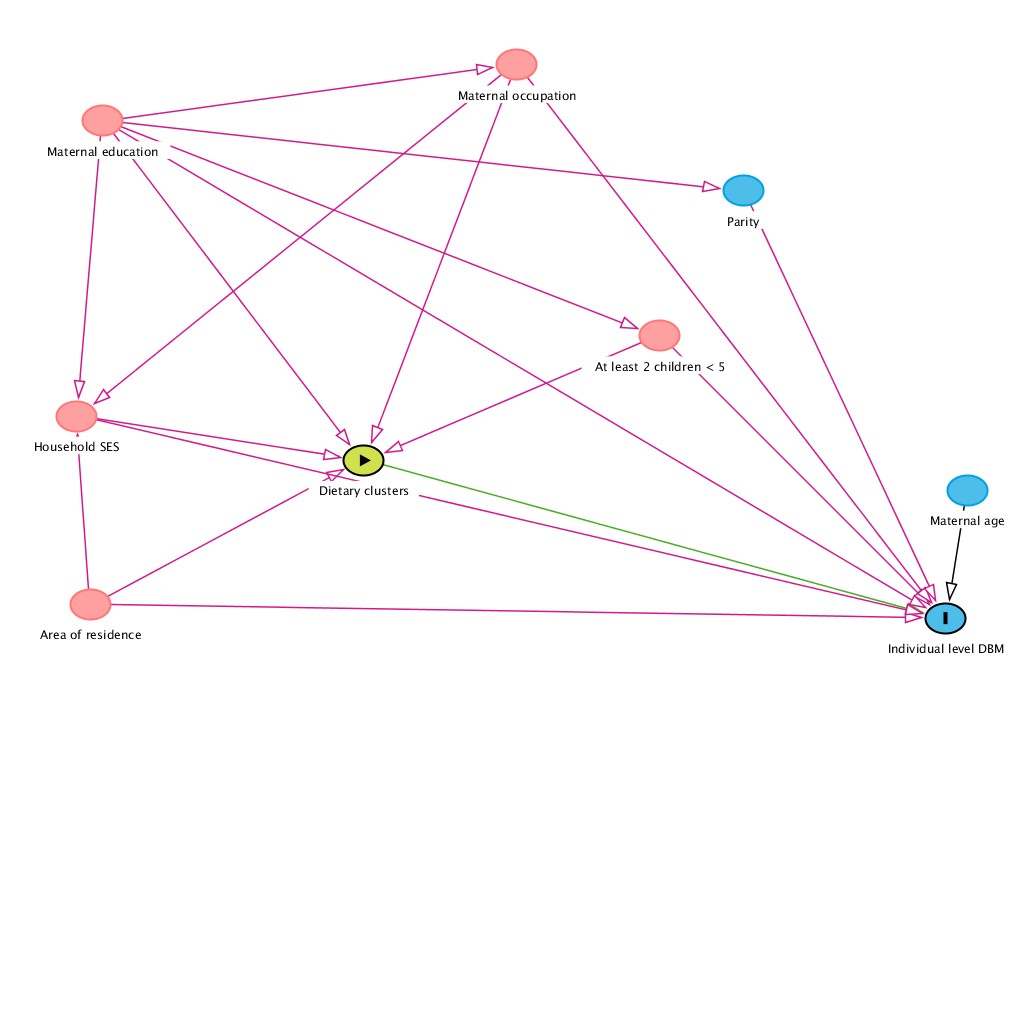


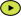
exposure
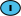
outcome
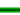
causal path
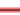
biasing path
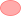
ancestor of exposure and outcome
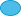
ancestor of outcome
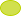
ancestor of exposure
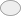
 unobserved

**Supplemental Figure 3**. Directed acyclic graphs (DAG) exploring the relationship between explanatory factors and dyad level double burden of malnutrition

*Model 1: DAG exploring the relationship between area of residence and dyad level DBM*


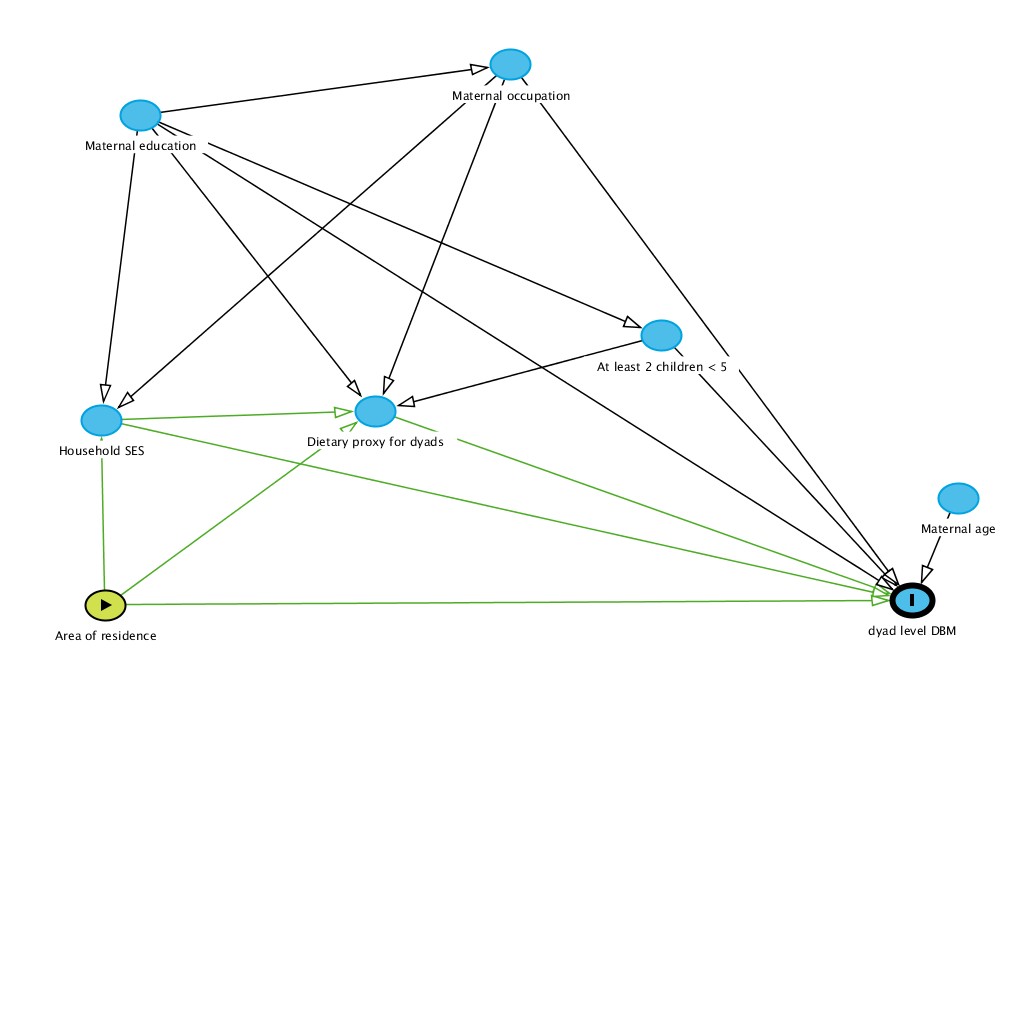


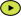
exposure
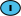
outcome
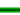
causal path
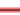
biasing path
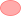
ancestor of exposure and outcome
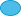
ancestor of outcome
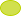
ancestor of exposure
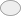
 unobserved

*Model 2: DAG exploring the relationship between household SES and dyad level DBM*


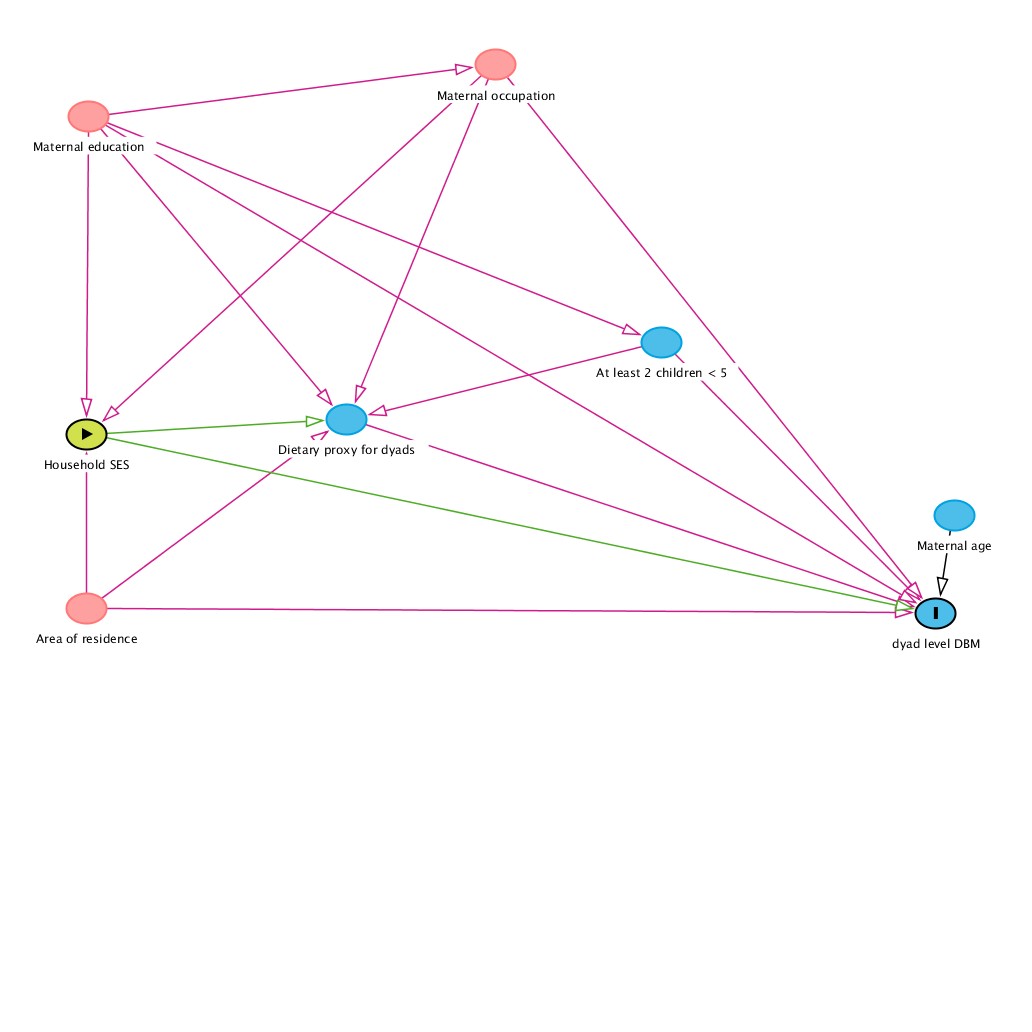


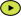
exposure
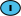
outcome
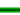
causal path
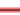
biasing path
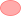
ancestor of exposure and outcome
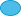
ancestor of outcome
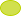
ancestor of exposure
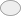
 unobserved

*Model 3: DAG exploring the relationship between maternal age and dyad level DBM*


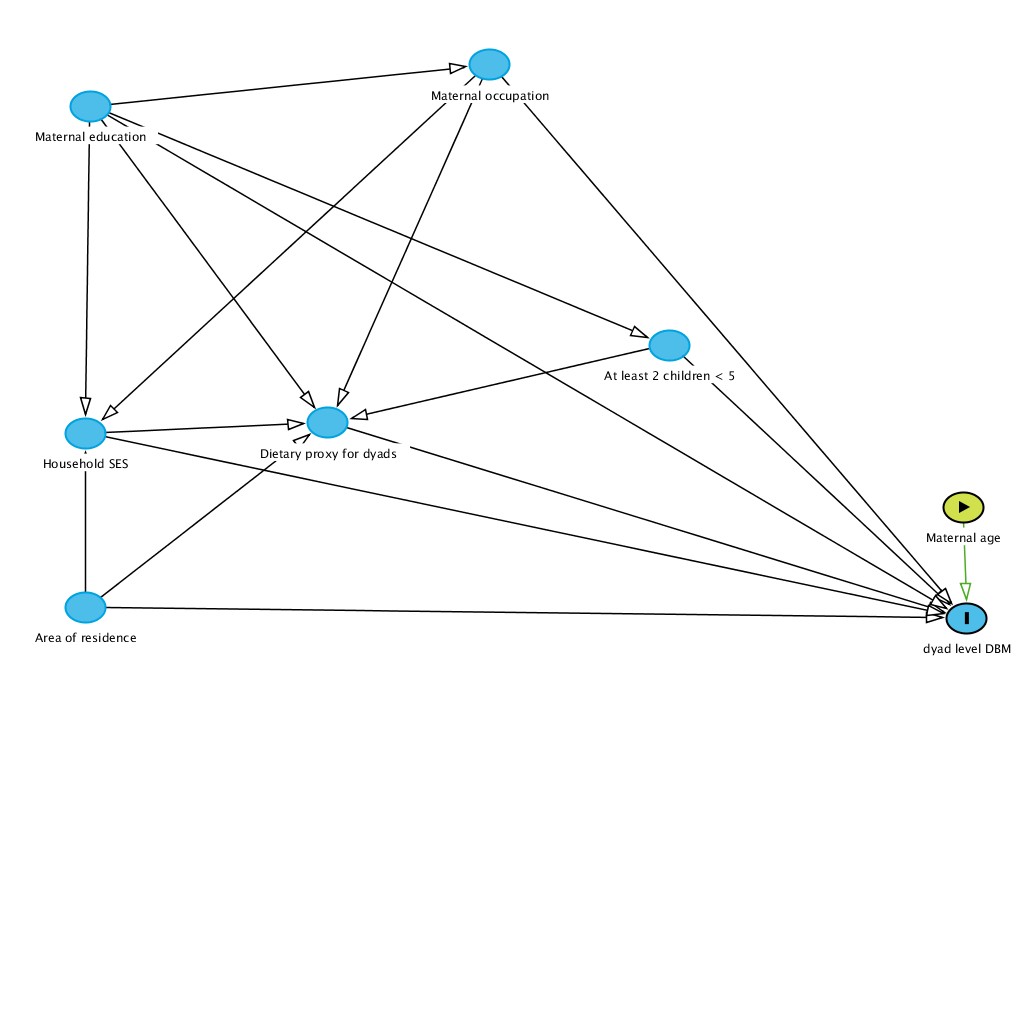


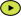
exposure
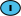
outcome
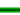
causal path
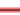
biasing path
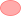
ancestor of exposure and outcome
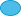
ancestor of outcome
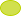
ancestor of exposure
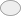
 unobserved

*Model 4: DAG exploring the relationship between maternal education and dyad level DBM*


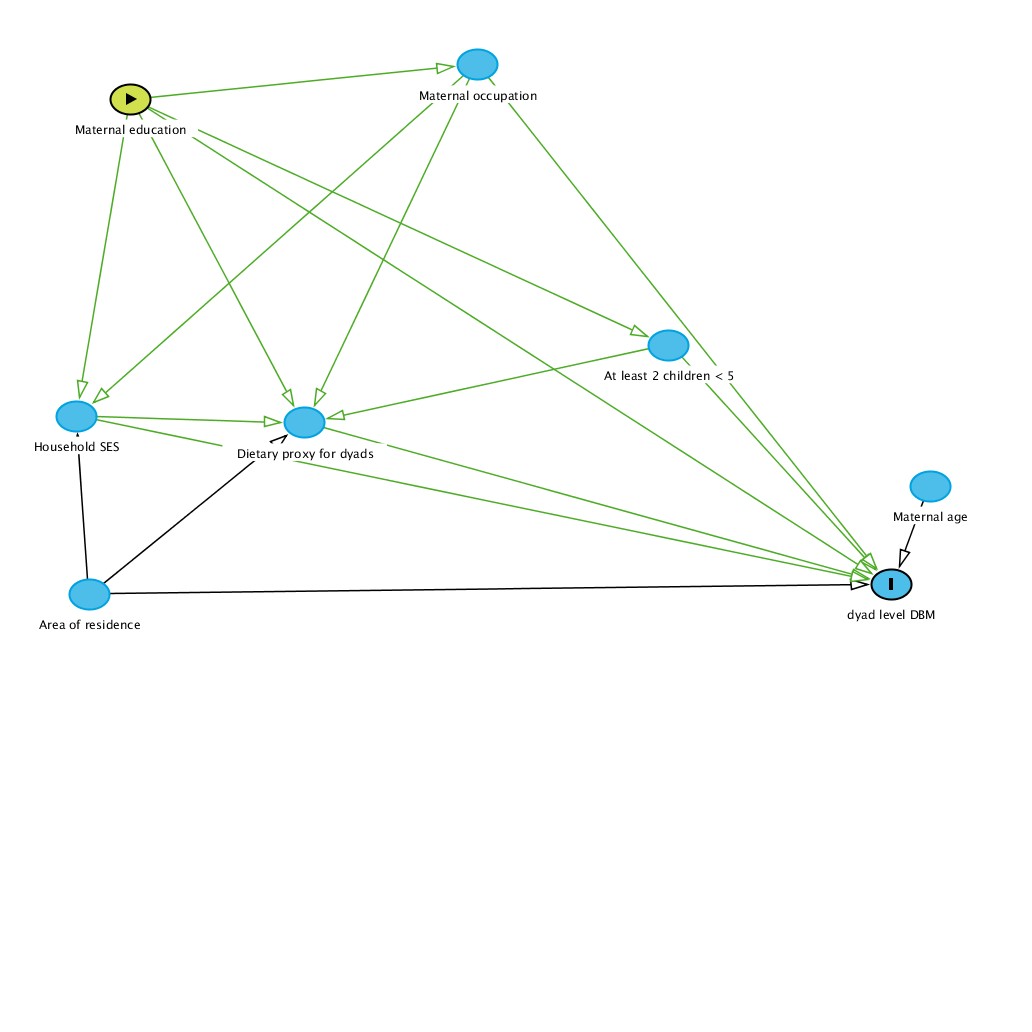


exposure outcome causal path biasing path ancestor of exposure and outcome ancestor of outcome ancestor of exposure unobserved

*Model 5: DAG exploring the relationship between maternal occupation and dyad level DBM*

exposure outcome causal path biasing path ancestor of exposure and outcome ancestor of outcome ancestor of exposure unobserved

*Model 6: DAG exploring the relationship between the number of children under five and dyad level DBM*

exposure outcome causal path biasing path ancestor of exposure and outcome ancestor of outcome ancestor of exposure unobserved

*Model 7: DAG exploring the relationship between the dietary proxy for the mother-child dyad and dyad level DBM*

exposure outcome causal path biasing path ancestor of exposure and outcome ancestor of outcome ancestor of exposure unobserved

**Supplemental Table 1.** Proxy indicators of the household environment, by area of residence

|  | Lima (n=125) | | Huánuco (n=119) | | Total | | P-value |
| --- | --- | --- | --- | --- | --- | --- | --- |
| Number of household members / room (3 categories) |  |  |  |  |  |  |  |
| *0-1* | 125 | 21 (16.8%) | 119 | 24 (20.2%) | 244 | 45 (18.4%) | 0.61 |
| *2-3* |  | 75 (60.0%) |  | 64 (53.8%) |  | 139 (57.0%) |  |
| *4+* |  | 29 (23.2%) |  | 31 (26.1%) |  | 60 (24.6%) |  |
| Flooring of the house (earth) | 125 | 9 (7.2%) | 119 | 34 (28.6%) | 244 | 43 (17.6%) | <0.001 |
| Ceiling of the house (cement) | 125 | 41 (32.8%) | 119 | 54 (45.4%) | 244 | 95 (38.9%) | 0.044 |
| Walls of the house (cement) | 125 | 26 (20.8%) | 119 | 31 (26.1%) | 244 | 57 (23.4%) | 0.33 |
| Sofa | 125 | 57 (45.6%) | 119 | 33 (27.7%) | 244 | 90 (36.9%) | 0.004 |
| Cupboard/Buffet | 125 | 44 (35.2%) | 119 | 22 (18.5%) | 244 | 66 (27.0%) | 0.003 |
| Wardrobe | 125 | 114 (91.2%) | 119 | 86 (72.3%) | 244 | 200 (82.0%) | <0.001 |
| Clock | 125 | 11 (8.8%) | 119 | 30 (25.2%) | 244 | 41 (16.8%) | <0.001 |
| Radio | 125 | 65 (52.0%) | 119 | 71 (59.7%) | 244 | 136 (55.7%) | 0.23 |
| Television | 125 | 121 (96.8%) | 119 | 109 (91.6%) | 244 | 230 (94.3%) | 0.081 |
| Cable TV | 125 | 100 (80.0%) | 119 | 46 (38.7%) | 244 | 146 (59.8%) | <0.001 |
| Food processor/blender | 125 | 91 (72.8%) | 119 | 84 (70.6%) | 244 | 175 (71.7%) | 0.70 |
| Microwave | 125 | 25 (20.0%) | 119 | 15 (12.6%) | 244 | 40 (16.4%) | 0.12 |
| Refrigerator | 125 | 95 (76.0%) | 119 | 67 (56.3%) | 244 | 162 (66.4%) | 0.001 |
| Washing machine | 125 | 44 (35.2%) | 119 | 12 (10.1%) | 244 | 56 (23.0%) | <0.001 |
| Computer | 125 | 15 (12.0%) | 119 | 28 (23.5%) | 244 | 43 (17.6%) | 0.018 |
| Internet | 125 | 17 (13.6%) | 119 | 20 (16.8%) | 244 | 37 (15.2%) | 0.49 |
| Bicycle | 125 | 16 (12.8%) | 119 | 5 (4.2%) | 244 | 21 (8.6%) | 0.017 |
| Car | 125 | 19 (15.2%) | 119 | 12 (10.1%) | 244 | 31 (12.7%) | 0.23 |
| Toilet facility in the home | 125 | 53 (42.4%) | 119 | 56 (47.1%) | 244 | 109 (44.7%) | 0.46 |

**Supplemental Table 2.** Exploring factors associated with maternal and dyad level double burden of malnutrition: univariate regression models

*Table 2a: Exploring factors associated with maternal double burden of malnutrition*

|  |  |  |  |
| --- | --- | --- | --- |
|  | OR | 95% CI | P-value |
|  |  |  |  |
| Area of residence: Huánuco (vs. Lima) | 0.85 | [0.44; 1.63] | 0.617 |
| Household SES |  |  | 0.934 |
| *Low* | ref |  |  |
| *Middle* | 1.09 | [0.48; 2.45] |  |
| *High* | 1.16 | [0.52; 2.59] |  |
| Maternal age (per five years) | 1.35 | [1.07; 1.71] | 0.013 |
| Maternal education ≥ secondary | 0.88 | [0.44; 1.74] | 0.706 |
| Maternal occupation (working) | 1.98 | [1.02; 3.88] | 0.045 |
| At least 2 children < 5 | 1.63 | [0.76; 3.49] | 0.208 |
| Parity | 1.28 | [1.05; 1.55] | 0.014 |
| Dietary clusters |  |  |  |
| *Cluster 1 "high variety, high sugary foods and beverages"* | 0.52 | [0.27; 1.01] | 0.053 |
| *Cluster 2 "high potato, low fruit and vegetables, low red meat"* |  |  |  |

*Table 2b: Exploring factors associated with dyad level double burden of malnutrition*

|  |  |  |  |
| --- | --- | --- | --- |
|  | OR | 95% CI | P-value |
|  |  |  |  |
| Area of residence: Huánuco (vs. Lima) | 0.42 | [0.24; 0.74] | 0.003 |
| Household SES |  |  | 0.410 |
| *Low* |  |  |  |
| *Middle* | 1.13 | [0.57; 2.22] |  |
| *High* | 1.55 | [0.79; 3.01] |  |
| Maternal age (per five years) | 1.41 | [1.15; 1.73] | 0.001 |
| Maternal education ≥ secondary | 0.56 | [0.32; 0.99] | 0.047 |
| Maternal occupation (working) | 1.81 | [1.02; 3.22] | 0.042 |
| At least 2 children < 5 | 2.42 | [1.25; 4.71] | 0.009 |
| Dietary indicator (proxy) for the mother/child dyad |  |  |  |
| *Cluster 1 "high variety, high sugary foods and beverages" + MDD child met* | 0.75 | [0.44; 1.29] | 0.303 |
| *Less optimal diet (i.e., any other combinations)* |  |  |  |
